# Mesenchymal stem cells from umbilical cord tissue as potential therapeutics for cardiomyodegenerative diseases – a review

**Published:** 2012

**Authors:** Trixi Hollweck, Christian Hagl, Günther Eissner

**Affiliations:** Department of Cardiac Surgery, Munich University Medical Center, Munich, Germany.

**Keywords:** Umbilical cord tissue, mesenchymal stem cells, cardiac differentiation, non-degradable scaffolds, polytetrafluorethylene, titanium, bioreactor

## Abstract

Heart failure is one of the leading causes of death worldwide. End stage disease often requires heart transplantation, which is hampered by donor organ shortage. Tissue engineering represents a promising alternative approach for cardiac repair. For the generation of artificial heart muscle tissue several cell types, scaffold materials and bioreactor designs are under investigation. In this review, the use of mesenchymal stem cells derived from human umbilical cord tissue (UCMSC) for cardiac tissue engineering will be discussed.

In humans, the heart is one of the least regenerative organs in the body (1). The limited ability of the heart to regenerate damaged tissue after major cardiac injuries often leads to heart failure (2). Despite a wide range of therapeutic approaches, heart failure remains the leading cause of death in modern societies (3,4). Myocardial infarction is the major cause of heart failure. Ischemic conditions result in an irreversible loss of functional cardiomyocytes which are gradually replaced by fibroblasts, forming non-contractile scar tissue (5). Resident cardiac progenitor cells can be found in transplanted human hearts, and evidence of myocyte proliferation in the human heart exists. However, this proliferation does not compensate for up to 1 billion cardiomyocytes being lost after MI (6). In end stage heart failure allogeneic heart transplantation remains the last treatment option, but it is limited due to donor organ shortage. According to the Eurotransplant International Foundation, in 2011 the demand for donor hearts was covered only to 35 % in Germany (7). The generation of artificial heart muscle tissue using cardiac tissue engineering might be a reasonable alternative to heart transplantation.

**Cardiac tissue engineering**

Cardiac tissue engineering is an interdisciplinary research area in regenerative medicine. Besides paracrine effects supporting angiogenesis, modulation of extracellular matrix components, and stimulating interactions with resident cardiac progenitor cells, the main aim of tissue engineering is the repopulation of the diseased myocardium with cells that can restore contractility (8-11).

**Cell application**

The route of administration of autologous and allogeneic cells is one of the central questions in cardiac tissue engineering. Cellular cardio-myoplasty is performed by intracoronary injection or direct implantation of a single cell suspension into the myocardium (12). Animal studies demonstrate an increase in the pumping function of the heart. However, myocardial regeneration was not observed (13). Functional improvement could be explained by secretion and stimulation of angiogenic growth factors resulting in the lack of myogenesis stimulation and contractility improv-ement (14). Systemic application also carries the risk of pulmonary accumulation of cells. Experimental injection of cells into the infarcted region ensures the delivery to the damaged area but is hampered by significant cell loss (12, 15).

An alternative approach to injection of isolated cells into the heart is the use of artificially engineered tissues that are geometrically, structurally and functionally defined prior to transplantation. Scaffolds are populated *in vitro* with cells and subsequently implanted onto the infarcted zone to allow precise cell delivery and mechanical support (4, 16). 

Resident cardiac stem cells can thus be stimulated to migrate into the area of regeneration induced by growth factors released from the implanted cells. Reconstitution of heart muscle tissue would also be possible by implanted cells themselves, differentiated *in vivo* into cardio-myocytes by local tissue-specific mechanism or differentiated *in vitro* prior to transplantation. In contrast to cell injection, using artificial heart tissue might results in less cell loss due to cell immobilization on scaffolds by adhesion molecules (17).

For myocardiac regeneration, cells from several cell sources like skeletal muscle (18) or neonatal rat heart (19) have been investigated already. Although some of these cell types integrate into damaged myocardium, application is restricted by limited availability and poor proliferation capacity (20). This has led to the search for alternative more efficient cell populations.

**Cell sources**

Heart muscle regeneration requires cells with the capability for proliferation, plasticity and functional integration into cardiac tissue (21). Stem cells feature unique regenerative potential and are consequently qualified for this claim (22-24). Due to their origin, stem cells are categorized into embryonic, induced and adult stem cells (25). Embryonic stem cells (ESC) derived from early embryos are well expandable and able to differentiate into various tissues. This pluripotency qualifies them for therapeutic applications, however ethical and legal concerns about using embryos for stem cell isolation exist. Moreover, in animal studies teratocarcinomas are described after implantation of ESC (26, 27). Induced pluripotent stem cells (iPSC) are thought to be an alternative cell source without ethical concerns (28). Since the discovery of genetic reprogramming of adult fibroblasts into pluripotent stem cells (29, 30) extensive efforts aim at the clinical applicability of iPSC, including reprogramming of fibroblasts using recombinant proteins (protein-induced pluripotent stem cells (p-iPSC)) (31).

Potential cardiomyogenic differentiation of p-iPSC also offers an option for cardiac tissue engineering. Other sources include hematopoetic stem cells and mesenchymal stem cells, either obtained from newborn, children or adults. They are collectively termed adult stem cells or postnatal stem cells, particulary if they are derived from infantile organisms (32). Mesenchymal stem cells (MSC) have the capability for self-renewal and differentiation into various lineages of mesenchymal origin, nerve and myogenic cells. Besides a comparable differentiation capacity, MSC seem to be more efficacious in tissue reconstitution than adult hematopoetic stem cells, due to strong pro-angiogenic properties necessary for a functional myocardium (33).

Moreover, MSC show a higher homing potential towards tissue defects resulting in the production of repairing growth factors (34, 35). Since they have the ability to differentiate into cardiomyocyte (36), MSC are a potential cellular source for cardiac stem cell-based therapy (35, 37). MSC have been already tested clinically and do not raise any ethical concerns (38). To date, human bone marrow (BM) represents the major source of MSC. However, aspirating BM from the patient is an invasive procedure and the number as well as the differentiation potential and the maximum life span of human BM-derived MSC (BMMSC) signi-ficantly decline with donor age (39, 40).

The umbilical cord tissue may be an attractive alternative source to BM (41). The two arteries and the single vein of the umbilical cord with a length up to 60 cm are surrounded with fetal connective tissue – the so called Wharton`s jelly, protecting the vessels against compression, torsion and bending (42). In line with several publications, Weiss et al. described MSC derived from the perivascular and intervascular region of the umbilical cord tissues (fig. 1), collectively termed umbilical cord tissue derived mesenchymal stem cells (UCMSC) (43-46).

In contrast to BMMSC, UCMSC are easily attainable and can extensively be expanded and maintained in culture, even after cryopreservation (43, 47-49). With regard to future clinical trials, our group successfully managed to grow UCMSC under GMP-compliant culture conditions, while retaining their phenotypic and functional properties (50). Due to close relation to the fetal phase, it is assumed that UCMSC are less determined than adult stem cells, show less teratogenic potential and are free of viruses (43, 51). In addition, UCMSC qualify for an allogeneic use due to their immunological naivity (51) and weaker response to inflammatory stimuli. With regard to their multipotency (32, 41, 51-52) UCMSC can be differentiated into bone, cartilage, neural and muscle cells as well as cardiomyocyte-like cells, as they express cardiac troponin-I and *N*-cadherin (32, 41, 43, 51-53).

**Fig 1:**
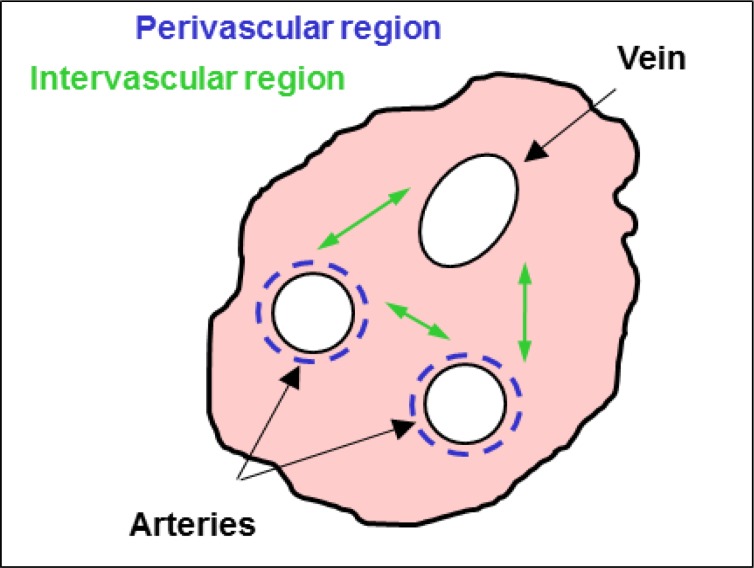
Umbilical cord profile. The two arteries and the single vein of the umbilical cord are surrounded by Wharton`s jelly, containing mesenchymal stem cells in the perivascular and intervascular region

**Cardiomyogenic differentiation**

Cardiomyogenic differentiation is presumed to be triggered by an increased expression of the embryonic transcription factor GATA-4 (54). GATA-4 proteins are not only important for heart development, but also constitute one of the earliest cardiac markers (55). In adult hearts, GATA-4 regulates the expressions of several sarcomeric proteins, which are used in combination with proteins of the troponin complex for the verification of induced cardiomyocytes (56). In addition, electric coupling by gap junctional connexins is essential for contraction (57). Contraction of cardiomyogenically differentiated stem cells of embryonic and adult origin has already been described, however with a percentage of contracting cells less than 10 % (58-60). 

Cardiomyogenic differentiation of BM-MSC and a significant improvement of left ventricular function after application of BM-MSC have also been published (61-63). However, the broad differentiation potential of MSC could also lead to undesirable effects. Since undifferentiated MSC tend to spontaneously differentiate into multiple lineages when transplanted in vivo, it is possible that such uncommitted stem cells undergo maldifferentiation within the infracted myocardium with potentially life-threatening consequences, e.g. osteogenic differentiation of BM-MSC within ischemic myocardium in a murine model (64). Although such phenomenons are not yet described for UCMSC (49) it was postulated that a certain cardiac differentiation of stem cells prior to transplantation would result in enhanced myocardial regeneration and recovery of heart function (65). 

In this context, initiating the transformation of stem cells into a cardiomyogenic lineage is accomplished by defined culture conditions (66). Embryo-like aggregates (67), the DNA demethylating agent 5-azacytidine (53, 68-69), several growth factors and the oxytocic hormone (70) are used to induce myocyte differentiation of various stem cell types. Maltsev *et al.* demonstrated the expression of cardio-specific genes, proteins and action potentials in cells differentiated from murine embryonal stem cells by cultivation in hanging drops as “embryoid bodies” (67). Using this differentiation system, UCMSC form aggregates, but cellular outspread is not sufficient for performing extensive analyses. Failure of cellular outgrowth may be explainable due to the dependence of this method on the initial cell number present in the aggregates (71). 

Based on a yet unknown mechanism, cytostatic 5-azacytidine results in cardiac differentiation of stem cells by DNA-demethylation (72). Cardiac differentiation of MSC induced by 5-azacytidine is controversially discussed. Martin-Rendon and colleagues report that 5-azacytidin treated human MSC derived from umbilical cord and bone marrow do not generate cardiomyocytes in vitro at high frequencies (23). 

In contrast, results of Antonitsis *et al.* and Pereira *et al.* indicate that adult human bone marrow MSC (73) and MSC from umbilical cord (35) can differentiate towards a cardiomyogenic lineage after 5-azacytidine treatment. These discrepancies might be explained by the variability in culture conditions (74) or by different specification criteria for what makes a cell a cardiomyocyte. For example, the use of cytokines and growth factors is a step forward in the development of a defined culture milieu for directing the cardiomyogenic differentiation. In this context, TGF-β and bFGF are the most important growth factors in embryonic cardiac development affecting cell proliferation, migration and differentiation (75). Xu *et al.* stated that bFGF is necessary during the differentiation process because of its capability to develop the myogenic phenotype and promote the formation of myotubes (76).

However, our study showed that UCMSC exposed to 5-azacytidine convert into cells changing their morphology and expressing cardiac-specific proteins irrespective of the presence of bFGF (71). 

UCMSC differentiated according to Wu *et al. *(69), using 5 µM 5-azacytidine for 24 h and bFGF containing culture medium, increase in size with striate pattern and express cardiac actin, cardiac actinin, sarcomeric actin, sarcomeric actinin, myosin heavy chain as well as connexin 43 after 5 weeks of culture. UCMSC treated with 3 µM 5-azacytidine for 24 h according to Wang *et al.* (53) and 10 µM 5-azacytidine for 72 h according to Matsuura protocol I (70) also change their morphology and express these cardiac specific proteins known for regulating contraction and gap-junctional communication without supplemented bFGF. TGF-β1 in combination with 5-azacytidine have been found to promote differentiation of human cardiomyocyte progenitor cells (68).

However, in our hands, the combination of 5-azacytidine and TGF-β1 stimulation of UCMSC leads to a flattened appearance and the expression of cardiac actin, cardiac actinin, sarcomeric actin, sarcomeric actinin as well as connexin 43 after 5 weeks of culture, but UCMSC do not express any troponins or myosins necessary for contraction. In addition, during the differentiation process, cell numbers decreased to levels insufficient for immunocytochemical analyses (71).

Cardiac differentiation of embryonic P19 carcinoma cells and adult Sca-1+ cells of murine heart is also described after exposure to oxytocin, the mechanism of action, however, is unknown (70-77). Oxytocin, a female reproductive hormone, is necessary for uterine contractions during ovulation and parturition. The expression levels of oxytocin are higher in developing hearts than in adult hearts suggesting that oxytocin may be involved in cardiomyocyte differentiation (78). Data from Matsuura *et al.* indicate that oxytocin is a more potent inducer of cardiac differentiation of Sca-1+ adult murine heart cells than 5-azacytidine (70). This is supported by our results figure 2, demonstrating that human UCMSC exposed to 10nM oxytocin for 72 h express the cardiomyocyte-associated proteins including cardiac actin (fig. 2a), sarcomeric actin (fig. 2b), cardiac troponin T (fig. 2d), connexin 43 (fig. 2e) cardiac actinin (fig. 2f), sarcomeric actinin (fig. 2g), and myosin heavy chain (fig. 2h) in significantly higher frequencies than after 5-azacytidine treatment (71). This analysis revealed that UCMSC can be differentiated into cardiomyocyte-like cells, however, functional analyses of oxytocin-differentiated UCMSC, such as to monitor action potentials, have yet to be performed.

**Scaffolds**

It is known that isolated cells are generally not able to form new tissue autonomously (79). For generating tissue *in vitro*, cells have to be colonized onto natural or artificial scaffolds. However, development of functional tissue requires an optimal interaction of cells and scaffolds. A scaffold should provide chemical stability and physical properties matching the surrounding tissue to provide cytocompatibility, support adhesion, proliferation, and mechanical strength (80). Additionally, scaffolds are required to retain cell phenotype and ensure protein synthesis (81). 

**Fig 2:**
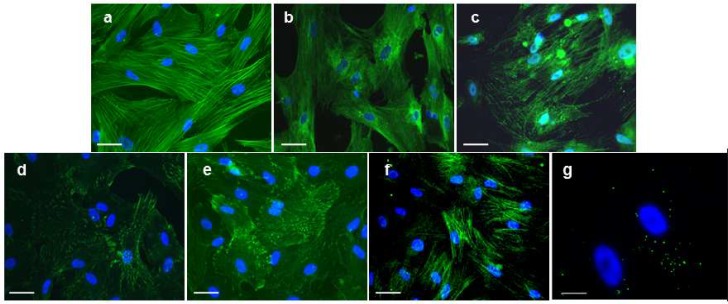
Immunocytochemical analysis of UCMSC differentiated according to Matsuura et al. using oxytocin (70). Cardiac differentiated UCMSC express the contractile proteins cardiac actin (a), sarcomeric actin (b), cardiac troponin T (c), cardiac actinin (d), sarcomeric actinin (e), myosin heavy chain (f) as well as the gap junctional protein connexin 43 (g) (a-g; all in green fluorescence) for electrical cell-to-cell coupling. Cell nuclei were stained by DAPI (a-g; blue) (71). Scale bars: a-f = 50 µm, g = 25 µm

In order to avoid implant rejection and inflammatory response, scaffolds should be biocompatible and sterilizable. For generating functional heart muscle, scaffolds should be made of flexible and tear-resistant material to allow contraction (80, 82). Regardless of which biocompatible material scaffolds are made of, the microarchitecture including porosity, pore geometry and the surface micro-texture considerably influence cell function (83). Both, scaffolds of biological origin and those made of synthetic material as well as some type of hybrids are currently under investigation for tissue engineering applications (84-86).

**Biomaterials**

*Decellularised tissues*

These tissues of allogeneic or xenogeneic origin are derived from enzymatic or detergent decellularisation (87). Cell-free tissues consist of natural extracellular matrix (ECM), degrade after implantation and are replaced by ECM-proteins of transplanted cells after re-seeding or by in-growing cells (88). Due to excellent mechanical properties, decellularised tissues are used for the development of viable heart valve prostheses (89). However, decellularisation can damage scaffold tissue, resulting in a decreased tensile strength and elasticity. Xenogenic decelluarised tissues undergo aneurysms and lead to infections and thrombosis. In addition, decellularisation might affect seeding efficiency due to residual antigenic components inducing humoral responses (87, 90, 91). 

*Biopolymers*

Natural polymers include fibrin, collagen, chitin, hyaluronic acid, and alginates. Besides enzymatic degradation, biopolymers show low inflammatory activity and toxicity (81). In addition, they support cell growth on implants due to their high protein content and accelerate healing because of strong adhesion to recipient organs (58). Fibrin is part of the blood clotting system and plays a central role in wound healing. As an alternative to conventional surgical sutures, fibrin glue is clinically used for wound closure (90). Collagen is another example of biopolymers in clinical practice. It is the predominant protein in the human body and the main component of ECM (81, 90). In cardiovascular surgery, collagen is used for heart valve replacement and blood vessel substitutes as well as for bone repair and burn and ulcer treatment (82). Naturally occurring biomaterials may most closely simulate the native cellular milieu, but large batch-to-batch variations upon isolation from biological tissues and poor mechanical strength are the main limitations for a clinical application. In addition, biopolymers are often denatured in a way no longer enabling tissue formation and often require chemical modifications, which can lead to toxicity (79, 81). 

**Synthetic materials**

*Degradable Polymers*

Numerous synthetic and degradable polymers like poly(α-hydroxy ester), particulary polyglycolic acid (PGA) or polylactid acid (PLA), polyanhydrides, polyorthoester and polyphosphazanes have been developed to overcome the limitations of natural materials mentioned above (79). Synthetic degradable polymers undergo degradation during cell culture or after implantation upon formation of tissue specific neo-ECM. Most of these polymers are resistant to enzymatic digestion, they are rather chemically hydrolyzed resulting in consistent and patient-independent degradation (79, 90).

In order to allow tissue generation and remodeling processes, microstructure, mechanical properties and resorption rate can be regulated by porosity and pore size, for example. After cell seeding, synthetic degradable polymers initially retain the cellular compound and ensure mechanical function of implants until an ECM is formed by colonized cells (90). However, if polymers degrade faster than the development of an ECM occurs, seeded cells lose their connectivity resulting in final cell loss and an inhibition of therapeutic effects. In addition, polyesters release degradation products which affect biocompatibility. Moreover, polyesters are stiff materials suitable for load-bearing implants, but the minimal flexibility precludes their use for soft tissues like heart muscle. 

*Non-degradable polymers*

Synthetic, non-degradable polymeres are characterized by structural resistance, a special topography and a three-dimensional form with defined pore sizes. Polyethylen terepthalate (PET, Dacron®), polyurethane (PU), and expanded polytetrafluorethylene (ePTFE) dominate the graft market (92) because of their anti-thrombotic properties. PET is a semi-crystalline aromatic polyester built of woven or knitted multiple fibres. While woven PET grafts feature small pores, knitted implants exhibit larger pores supporting tissue ingrowth. PET is mainly used for artificial blood vessels, tendon substitutes and surgical sutures (82, 86). 

PU is a polymer with a characteristic urethane group. Within the monomeric unit, moieties could be substituted by different groups, resulting in versatile properties. Fabrication of hydrolytic stable PU led to the development of different implants like vascular grafts, artificial heart valves and catheters (82, 86). PTFE is an unbranched linear polymere built of fluorine and carbon. Expanded PTFE (ePTFE) features nodes in fibrillar structure with longitudinal internodal distances of 17-90 µm. Due to the symmetrical design of the monomeric unit, crystallinity of ePTFE come up to 94 % preventing degradation. Besides easy availability, ePTFE offer non-immunogenic and anti-thrombotic properties (86).

ePTFE is clinically used for cardiac, groin and vascular grafts. However, the use of synthetic non-degradable polymers as scaffolds for tissue engineering is often limited by the poor retention of cells to these hydrophobic biomaterials (82, 93-95, 96). Yu et al. report, that endothelial cells – in contrast to smooth muscle cells - adhere poorly to ePTFE. In our experiments, the investigation of adherence, viability, proliferation and morphology of UCMSC on uncoated ePTFE scaffolds showed poor results (97). This is in line with results from Neuss et al. (80), demonstrating that bone-marrow derived MSC (BMMSC) display a round, spherical morphology on ePTFE. Furthermore, ePTFE does not allow BMMSC proliferation, indicating that cells need an underlying matrix providing them with sufficient binding sites.

**Hybrids**

Hybrids combine advantages of different materials in one composite. Surface modification of non-degradable polymers increases the wettability leading to an improved seeding efficiency. The knowledge that positively charged surfaces are more conductive to cell adhesion and morphological maturation, led to the examination of various adhesive coatings of synthetic materials (98). The matrix molecules of these coating substances, such as albumin, collagen, fibrin, gelatine, fibronectin, laminin and fibrin glue, bind directly to specific domains on the cell membrane (95, 99). For example, Kaehler et al. and Feugier et al. pre coated ePTFE vascular prostheses with fibronectin-treated Type I/III collagen and reported a higher cell adherence and spreading on these grafts (100, 101). Although coating with specific proteins improves cell adhesion, the integrity of the coating is compromised by mechanical stress (94). Furthermore, if surfaces are not completely endothelialized or endothelial cells are lost upon exposure to mechanical loadings, these coatings attract platelets. In these cases, the technique leads to a more thrombogenic surface, which defeats the purpose of cell seeding (102). 

To overcome the limitation of biological coatings, a hydrophilic titanium-coated surface can be obtained by plasma-assisted chemical vapour deposition (PACVD): The resulting covalent bonding can only be separated by destroying the synthetic structure itself. The titanium layer is extremely thin and has the same flexibility as the synthetic material. Titanium-coated synthetics feature outstanding wettability enabling them to adapt to the anatomical environment and to enhance cell adhesion (92). Moreover, titanium-coated synthetic implants provide excellent biocompatibility because of the oxide layer which forms under atmospheric conditions. Therefore titanium and its alloys are widely used as biomaterials in association with tissue, bone, and blood (103-105). Our findings, that titanium-coated ePTFE figure 3a, scaffolds are superior to uncoated ePTFE scaffolds figure 3b, in UCMSC adherence, viability and proliferation (97) are in line with results of previous studies, demonstrating a support of MSC adhesion and proliferation on titanium dishes (104, 106).

**Bioreactors**

For the use of tissue engineered constructs *in vivo*, it is essential to examine their functionality and mechanical integrity prior to implantation (107). In addition, forces acting directly or indirectly on cells, e.g. via scaffolds, can affect cellular differentiation (82). *In* *vivo*, cells are stimulated continuously by mechanical, electrical and chemical signals influencing their phenotype, morphology and proliferation. If these signals are inappropriate or absent, cells lose their ability to form organized tissues (108). Thus, bioreactors simulating physiological conditions, such as mechanical shear stress, play a crucial role in the development of tissue engineered constructs (107).

The development of an effective bioreactor requires the consideration of various parameters. Ideally, bioreactors allow the regulation of physical parameters such as temperature, pH, pO_2_, pCO_2_, allow nutrient supply and removal of toxic metabolites as well as mechanical stimuli. Moreover, the material must be compatible with the manufacturing process, sterilization technique and the cultured cell type (109). Bioreactors can be applied for cell seeding, cultivation of colonized scaffolds and for conditioning of functional tissue engineered prostheses (110-112). 

In heart valve fabrication, bioreactors for tissue formation under dynamic culture conditions were demonstrated several times (107, 113, 114). Bioreactors also support tissue formation of heart muscle *in vitro* (115, 116). An effective approach to improve the contractile properties of artificial heart muscle constructs is electrical field stimulation or mechanical stimulation by unidirectional or auxotonic stretching (117). Accompanied by an improvement of contractile function, some studies demonstrated extracellular matrix formation, increased cell proliferation and uniform cell distribution of strained constructs (118,119). 

**Fig 3:**
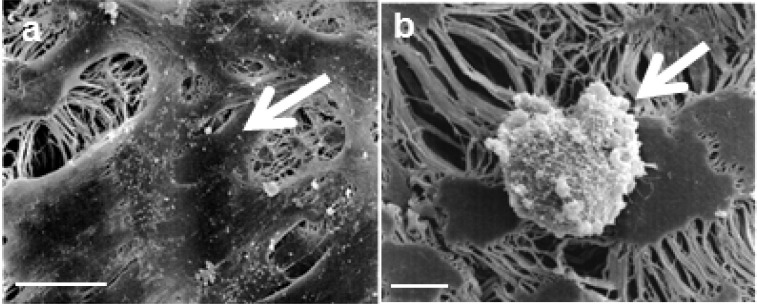
Morphology of UCMSC seeded on uncoated and titanium-coated ePTFE. UCMSC display their characteristic spindle-shaped morphology in a homogenous coverage on titanium-coated ePTFE (a, arrow) in contrast to a spherical morphology seeded on uncoated ePTFE (b, arrow) (97). Scale bars: a = 10 µm, e = 50 µm

**Fig 3:**
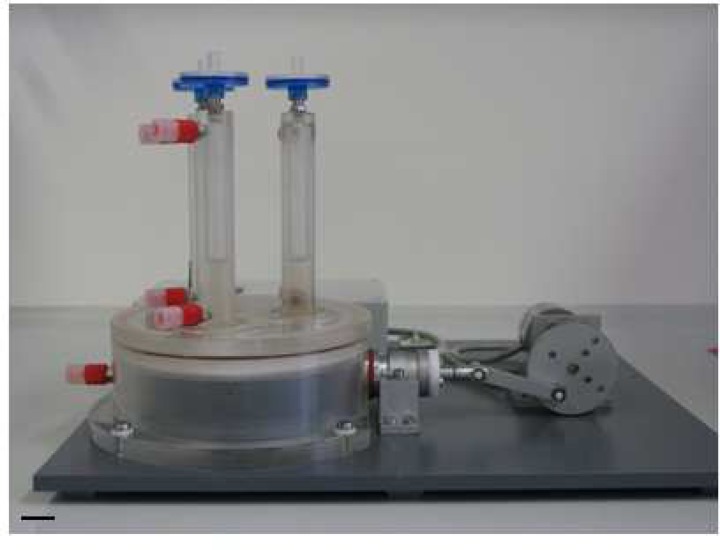
Morphology of UCMSC seeded on uncoated and titanium-coated ePTFE. UCMSC display their characteristic spindle-shaped morphology in a homogenous coverage on titanium-coated ePTFE (a, arrow) in contrast to a spherical morphology seeded on uncoated ePTFE (b, arrow) (97). Scale bars: a = 10 µm, e = 50 µm

In this context, Zimmermann et al. reported from highly differentiated cardiac tissue constructs after cyclic mechanostimulation in a stretch device (58). Sodian et al. developed a closed-looped perfused bioreactor by combining pulsatile perfusion and periodically stretching of tissue-engineered patch constructs (120). Birla et al. described a bioreactor system that applies electromechanical stretch to bioengineered heart muscle constructs with no evidence of physical damage (121). In order to repopulate ischemic myocardium with cells that might restore contractility, we analyzed the stability of the cellular coating upon mechanical stress in a newly developed bioreactor figure 4, mimicking myocardial contraction (122).

Three fluid compartments enable the comparison of different media, cells and scaffolds at defined mechanical loadings. Manufacturing of the core unit from acrylic glass provides optical transparency for macroscopical observation of processes within the unit. Elements of acrylic glass, stainless steel, Teflon® and silicone are robust and can be gas sterilized. Fixing of seeded scaffolds by clip-systems allows an easy assembling, reliable fixing and facilitates sterile handling. The speed controlled gear motor provides frequencies at 1-65 Hz, offering gradually increasing mechanical loadings of the tissue-engineered scaffolds. In addition, the bioreactor was designed in a dimension that allows its operation in a standard incubator. Preliminary experiments with UCMSC-seeded ePTFE scaffolds show the mechanical integrity of the cellular coating after friction stress generated in the pulsatile bioreactor. Viability and ultrastructural morphology of the stem cells are also maintained upon mechanical stress (122).

## Conclusion

Cardiac tissue engineering using UCMSC represents a promising approach for the repair of the injured heart, however, clinical relevance of tissue engineered constructs have to be evaluated *in vivo*. Functional regeneration of heart tissue after cardiomyodegenerative diseases should be demonstrated by the integration of UCMSC-seeded implants and/or their interaction with resident cardiac stem cells. In addition, survival of implanted constructs, tissue-specific differentiation and vascularization have to be verified. Moreover, electric integration resulting in functional reconstitution of the injured muscle tissue is a key step in the evaluation of safety and efficiency of UCMSC-seeded implants. 
